# Would the Addition of Immunotherapy Impact the Prognosis of Patients With Malignant Pericardial Effusion?

**DOI:** 10.3389/fonc.2022.871132

**Published:** 2022-05-06

**Authors:** Varsha Chiruvella, Asad Ullah, Islam Elhelf, Nikhil Patel, Nagla Abdel Karim

**Affiliations:** Medical College of Georgia, Augusta University, Augusta, GA, United States

**Keywords:** malignant pericardial effusion, combined chemoimmunotherapy, immunotherapy, adenocarcinoma, pneumonia

## Abstract

Pericardial effusion is a common finding in advanced-stage lung cancer. The presence of malignant cells or drainage of exudate effusion in the pericardial space may cause symptoms of dyspnea, pleuritic chest pain, and syncope. In addition to the difficulty physicians face in the detection and diagnosis of malignant pericardial effusion, treatment may be challenging considering the cancer prognosis and cardiovascular stability of the patient. Despite the availability of several treatment modalities for malignant pericardial effusion, including chemotherapy and surgery, patients with lung cancer historically present with poor prognoses. In addition to lung adenocarcinoma with malignant pericardial effusion, this case was complicated by COVID-19 and malignancy-associated obstructive pneumonia. We present a case of a 64-year-old woman with advanced non-small cell lung carcinoma (NSCLC) with malignant pericardial effusion who, despite testing positive for COVID-19 and having obstructive pneumonia, had favorable outcomes following systemic therapy with combined chemo-immunotherapy.

## Introduction

Malignant pericardial effusion (MPE) is a rare complication of advanced cancer in which cancer causes excessive fluid inside the pericardial sac, creating pressure on the heart and preventing it from pumping normally. While pericardial effusions may be non-malignant or malignant, malignancy accounts for up to 23% of pericardial effusions and 33% of symptomatic pericardial effusions (1, 2). Metastasis to the pericardium, resulting in effusion, is common in many heterogeneous types of cancers, with 5–15% of patients reportedly having malignant pericardial effusion (3, 4). However, only one-third of cancer patients with cardiac metastasis develop clinically significant MPEs that require intervention (5). Although there are many reported treatment modalities used in the management of MPEs, chemoimmunotherapy and immunotherapy are not commonly reported in the literature. MPEs in advanced-stage malignancies are also associated with high morbidity and mortality rates.

This case is unique because the patient presented with advanced stage IV NSCLC with MPE initially responded well to combination chemotherapy and immunotherapy. This case demonstrates the effective nature of chemo-immunotherapy for treating non-small cell lung cancer (NSCLC) in the presence of MPE. It also emphasizes the benefit of disease control despite being complicated by COVID-19 and pneumonia.

## Case Presentation

The patient in this case report was a 64-year-old female with stage IV metastatic non-small cell lung cancer, adenocarcinoma subtype, primarily in the right upper lobe with pericardial effusion. The patient had a history of pacemaker placement in 2008 due to sick sinus syndrome, non-insulin-dependent diabetes mellitus (NIDDM), tobacco dependence, and chronic systolic heart failure and was taking 81 mg aspirin daily. She initially presented to the hospital with the chief complaints of malaise and abdominal pain. She also admitted to occasional palpitations and a 20-lb weight loss over the past few weeks. Upon further investigation, chest radiography revealed a mass in the right upper lobe (RUL) mass. The patient was followed up with outpatient oncology but presented three days later to the emergency room with complaints of dyspnea in addition to a sore throat, intermittent diarrhea, and midsternal, non-radiating, non-pleuritic, non-reproducible, and non-exertional chest pain.

On examination of the dyspnea of the patient, she was normotensive with a blood pressure of 111/59 mmHg, tachycardia with a heart rate of 109 bpm, tachypnea with a respiratory rate of 31 bpm, and normal O2 saturation in room air.

The patient had already undergone extensive investigations at an outside hospital for dyspnea and chest pain, where the patient underwent chest radiography, which revealed a mass in the right upper lobe.

**Figure 1:**
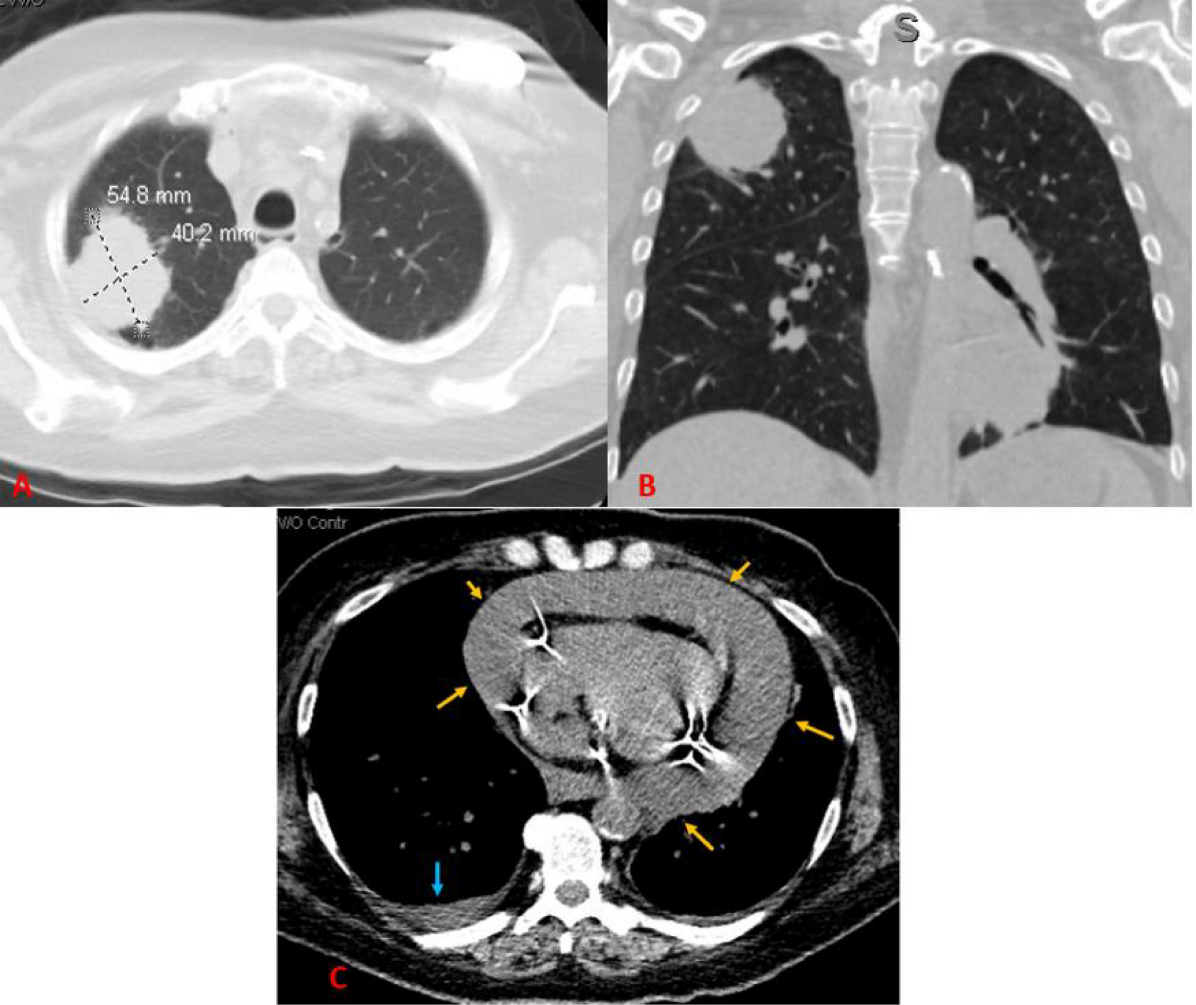
A CT scan of the chest without contrast. **(A)** Axial image showing a right upper lobe mass measuring approximately 5.5 × 4.0 cm in maximum axial dimensions. **(B)** Coronal image showing the right upper lobe mass contacting the pleura. **(C)** Axial image showing a large pericardial effusion (yellow arrows) and a small right pleural effusion (blue arrow).

Following this discovery, computed tomography (CT) of the thorax and abdomen/pelvis without contrast demonstrated a 5.5 × 4.0 cm RUL mass, which was highly suspicious for neoplasm. Mild mediastinal and right hilar lymphadenopathy were observed, suggestive of metastatic disease. A CT also revealed a large pericardial effusion (3.2 cm in greatest dimension) with no evidence of tamponade, a small bilateral pleural effusion, and a small number of ascites (**Figure 1**). CT of the head without contrast showed no evidence of acute intracranial abnormalities or metastatic disease. Because of the pacemaker of the patient, magnetic resonance imaging (MRI) was not performed.

**Figure 2:**
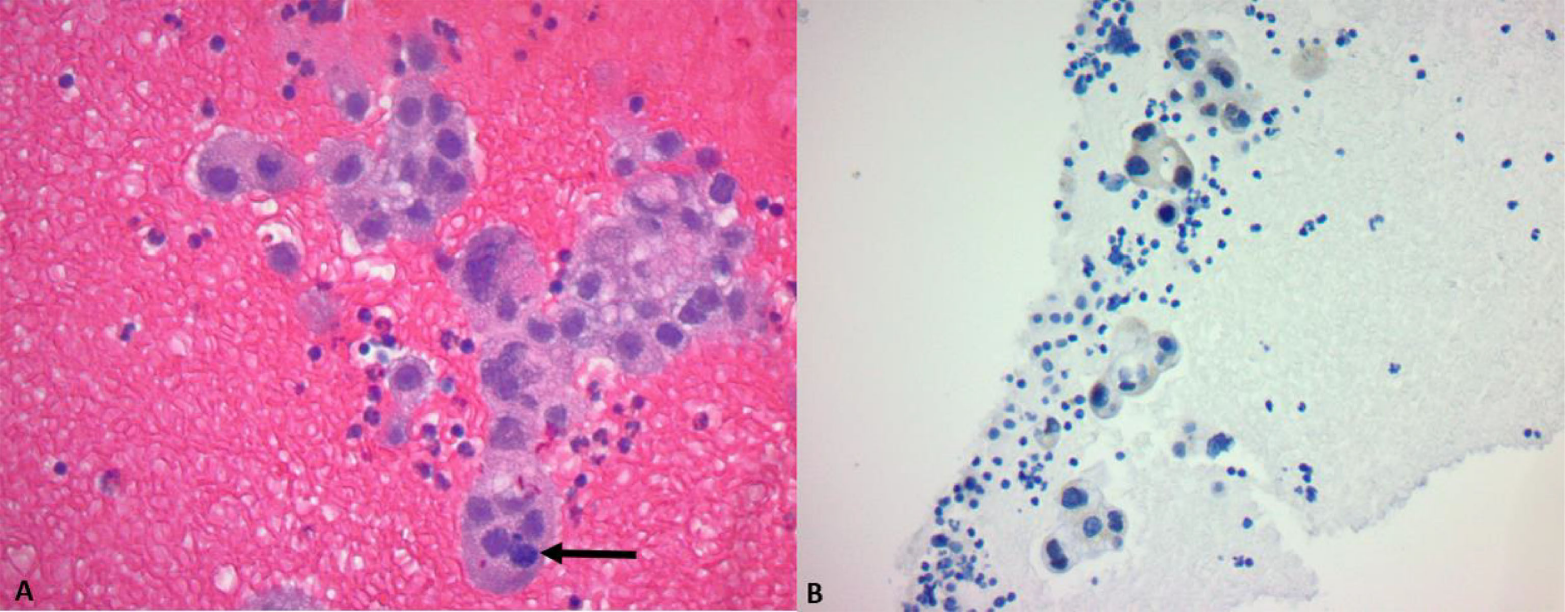
**(A)** H&E ×40: Poorly forming glands with vacuolated cytoplasm, hyperchromatic and pleomorphic nuclei, and an identifiable mitosis (arrow). **(B)** TTF1 stain: a cluster of tumor cells shows nuclear positivity.

A subxiphoid pericardial window was performed owing to the presence of a large (3.2 cm in diameter) effusion. The fluid was collected for cytology and a tissue biopsy was taken for histological examination. Histological examination of the pericardial tissue showed irregular clusters of poorly forming glands with eosinophilic, vacuolated cytoplasm, hyperchromatic, pleomorphic nuclei with identifiable mitosis in the background of red blood cells, and small lymphocytes. The tumor cells were positive for thyroid transcription factor 1 (TTF1). Based on histology and the pattern of immunohistochemical staining, the diagnosis of adenocarcinoma was made (**Figure 2**). The pericardial fluid was also positive for malignant cells. Based on radiologic and histological examination, the disease of the patient was staged as T3N2M1a. non-small cell lung carcinoma (NSCLC).

Molecular profiling of the tumor cells indicated high expression (>50%) of the PD-L1 marker and the genomic variants TP53, NRAS, and P5469V BRAF.

During systemic therapy, the patient tested positive for the B.1.351 variant of COVID-19. After an infectious disease consultation, the patient was diagnosed with post-obstructive pneumonia secondary to lung malignancy, rather than SARS pneumonia, as the former was more consistent with her CXR and WBC count. Once the patient was clinically stable and ready for discharge, a PICC line was placed in each inpatient. Due to her positive COVID-19 status, the chemotherapy plan for the patient was delayed by 12 days and was set to begin after the patient completed self-quarantine.

Once her self-quarantine for COVID-19 was completed, the patient underwent a TTE that showed a stable/decreased size of pericardial effusion (1.1 cm at its greatest dimension). She reported feeling better than when she presented to the hospital the previous month. She denied orthopnea, dyspnea, or lower extremity swelling and could ambulate and perform all activities of daily living without assistance. She had been compliant with all her medications, including ceftriaxone and azithromycin for obstructive pneumonia and daily medications of 81 mg aspirin, atorvastatin, lisinopril, and metformin. The patient received a systemic combination of carboplatin/pembrolizumab/pemetrexed every three weeks for four cycles, followed by pemetrexed and pembrolizumab maintenance. The patient received this combination therapy every 3 weeks (1 cycle) for a total of four cycles with maintenance as she did not have any genomic alteration such as *EGFR*, *ALK*, *ROS*, *B-RAF*, *MET*, or *RET* to allow the use of FDA approved targeted therapies. Additionally, PD-L1 showed no expression. The patient had a markedly good response and was classified as having partial remission according to the Response Evaluation Criteria for Solid Tumors (RECIST) version 1.1 criteria, measurable by a >30% reduction in total tumor size.

She denied worsening cough, hemoptysis, weight loss, fatigue, abdominal pain, change in urinary or bowel habits, blurred vision, or headaches at recent follow-up visit,. Her appetite increased and she gained subsequent weight. Status-post maintenance pemetrexed/pembrolizumab maintenance therapy, she developed a grade III maculopapular rash and auto-immune hypothyroidism related to immunotherapy. Hypothyroidism is now controlled with Synthroid. Pembrolizumab was stopped due to side effects, and the patient is now on pemetrexed alone as maintenance therapy. Her recent scans show overall stable disease. The patient continues to have no evidence of disease progression, either clinically or radiologically.

## Discussion

Malignancies commonly associated with MPEs include lung and breast cancers, melanoma, lymphoma, and leukemia. Lung cancer is the leading cause of MPE, with 3% of lung cancer patients reportedly experiencing MPE with a subsequent poor prognosis (6). Non-malignant causes include idiopathic, viral, cardiac, autoimmune, and medication-induced diseases (i.e., cyclophosphamide, doxorubicin), radiation-induced, traumatic, and metabolic (7). The most common symptom of pericardial effusion is dyspnea, in addition to tachycardia, pleuritic chest pain, cough, fatigue, hoarseness, and syncope, which may be related to tamponade. Radiological assessment for MPE includes a chest X-ray (CXR) that would show an enlarged cardiac silhouette with clear lungs. The “water bottle sign” is the classic sign of pericardial effusion (8). Additionally, an electrocardiogram (ECG) may indicate nonspecific ST- or T-wave changes, electrical alternans, and low QRS voltage, indicative of tamponade commonly caused by malignant pericardial effusion (7, 9). Although 12-lead ECG findings of low voltage and electrical alternans suggest pericardial effusion, they are poorly diagnostic and nonspecific for pericardial effusion and cardiac tamponade (10, 11). Similarly, chest radiograph findings may suggest the presence of pericardial effusion, but the findings are nonspecific and cannot be considered diagnostic for the presence of pericardial effusion. The diagnostic approach, in addition to ECG and radiological assessment, consists of echocardiography, a practice consistent with the 2015 European Society of Cardiology guidelines for the diagnosis and management of pericardial disease (12).

MPE is associated with high morbidity and mortality, especially in patients with primary lung cancer (13). The overall prognosis of patients with malignant pericardial effusion is primarily influenced by the extent and histological features of the underlying cancer. In patients with lung cancer, pericardial effusion causes a median survival time of 3 or fewer months. In particular, MPE in patients with NSCLC functions as an independent prognostic factor of cancer death and offers significantly decreased overall survival, with the incidence of MPE approximating 2–3%. One study found that patients with advanced NSCLC with MPE had a lower median survival time of 7.6 months compared to patients without MPE (15.0 months) (14). Multivariate analysis showed that age >65 y, underlying lung cancer, platelet counts <20,000, and the presence of malignant cells in the pericardial fluid were independently associated with poor prognosis. It has also been shown that patients with malignant effusions have significantly shorter overall median survival compared with those with non-malignant effusions (1-year survival estimate of 16.2% vs 49.0%, respectively) (15). As lung cancer is the most common malignancy associated with pericardial effusion, one study found that patients with NSCLC had a poor 2-year survival rate of 8.9% (14). Gross et al. demonstrated that the median survival of symptomatic malignant pericardial effusion is between two and four months (16).

Echocardiography is essential for determining the size and location of pericardial effusion and determining pathophysiologic changes such as right-sided chamber collapse and changes in inspiratory pressure gradient during left ventricular filling. Once a patient is diagnosed with tamponade, pericardiocentesis is performed under echocardiography guidance. Generally, pericardiocentesis is the first-line treatment for acute pericardial effusion with tamponade. However, the pericardial window has been regarded as a better technique for infective or systemic disease or pericardial effusion recurrence (17). Through a subxiphoid approach, pericardiocentesis involves inserting a long 18–22 gauge needle attached to a syringe between the xiphisternum and left costal margin, directed toward the left shoulder with continual aspiration. In patients with MPE, a pericardial window may be preferred due to a lower risk of recurrence (18–20). However, in patients with lung cancer and MPE, studies have shown that the pericardial window may be associated with worse overall survival when compared to use in non-lung cancer patients (21).

Classic therapeutic modalities used for treating pericardial effusion include pericardiocentesis, systemic chemotherapy, radiotherapy, and surgical subxiphoid pericardial window surgery (22). Immediate relief is achieved with percutaneous drainage, a surgical pericardial window, and systemic chemotherapy (22–24). The determination of the appropriate treatment modality for a patient depends on the urgency, the likelihood of tumor response to anti-neoplastic treatments, and the anticipated survival of the patient. Studies have reported the efficacy of systemic chemotherapy as monotherapy and in combination with a surgical pericardial window or pericardiocentesis (25). One study found that the best responses and survival estimates were in lung and hematologic cancer patients treated with pericardial window formation with systemic chemotherapy (26). Although lung cancer is the primary malignancy associated with pericardial effusion and has the poorest prognosis, systemic chemotherapy has shown some efficacy in reducing mortality in NSCLC patients with MPE. According to the RECIST criteria, however, one study noted that out of 98 patients with various cancers such as breast, lung, stomach, and colon cancer, approximately 74 patients were classified as having progressive disease after one cycle of chemotherapy (27). In contrast, our patient was categorized as having partial remission (PR) based on the RECIST criteria after four cycles of chemo-immunotherapy. With the increasing use of immunotherapy and chemo-immunotherapy in the treatment and management of primary and metastatic malignancies, the way physicians approach MPE may involve the incorporation of targeted therapy.

However, current reports on the prognostic value and efficacy of chemoimmunotherapy for MPE in patients with malignancy are scarce. In fact, there have been several reports of immunotherapy as a cause of pericardial effusion and cardiac tamponade (28–33). Both nivolumab and pembrolizumab are checkpoint inhibitor (CPI) monoclonal antibodies against PD-1 proteins expressed on T cells, B cells, macrophages, and dendritic cells and have both induced pericardial effusions in patients with adenocarcinoma of the lung. Unlike cytotoxic chemotherapeutic agents, immune checkpoint inhibitors activate T cells. Therefore, their adverse effects are mostly immune-mediated reactions such as rash, colitis, hepatitis, thyroiditis, hypophysitis, pneumonitis, and pericarditis, which may progress to pericardial effusion and tamponade (34). Cancer patients beginning CPI immunotherapy with malignant involvement of visceral spaces should thus be closely monitored for CPI-induced, rapidly evolving pericardial and tamponade conditions, as these conditions may represent pseudoprogression (28). However, there are currently no studies on the efficacy of combining immunotherapeutic agents with chemotherapy for managing MPE. As far as we are aware, this is the first case to show an effective reduction in effusion size and a favorable survival outcome in a patient with lung adenocarcinoma that has spread to the pericardium.

Additionally, this case presents the benefit of chemoimmunotherapy in a patient with cancer who has a history of testing positive for COVID-19 and obstructive pneumonia. The COVID-19 pandemic has spread globally, resulting in more than 28 million positive cases, more than 500,000 deaths in the United States, and more than 2.4 million deaths worldwide as of 23 February 2021 (35). Studies have shown that among patients with COVID-19, those with cancer have worse outcomes than those without underlying malignancy (36, 37). It has also been shown that patients with lung cancer, particularly NSCLC, are at greater risk of mortality in the presence of COVID-19 (38–40). While studies on the efficacy of chemoimmunotherapy in NSCLC complicated by COVID-19 are limited, one case reports a NSCLC patient on a combination of carboplatin/pembrolizumab/pemetrexed who developed COVID-19 pneumonia and responded well to anti-interleukin 6 receptor tocilizumab (41). This report demonstrates the potential efficacy of immunotherapy in patients with NSCLC complicated by COVID-19. In addition to COVID-19, our patient was treated for obstructive pneumonia using ceftriaxone and azithromycin. Although many reports have shown an association between COVID-19 and pneumonia, thereby termed COVID-19 pneumonia, our patient was rather classified as having obstructive pneumonia secondary to NSCLC (42, 43). Nonetheless, a study reported an improvement in outcomes in COVID-19 lung cancer patients treated with a combination of hydroxychloroquine and azithromycin (36). These findings of improved outcomes associated with azithromycin and tocilizumab immunotherapy in lung cancer patients with pneumonia and COVID-19, respectively, could further explain the favorable clinical outcomes in our patient following treatment. More data from advanced-stage lung cancer patients with pericardial metastasis affected by COVID-19 must be collected to draw conclusions regarding the efficacy of immunotherapy in treating these complications.

COVID-19 has unsurprisingly caused disruption to standard cancer care, which includes systemic anticancer therapy (SACT). With many countries and states entering lockdown periods, the virus has caused variable rates of patient deferment and presentational and diagnostic delays, causing setbacks in accessing urgent evaluations and treatment for malignant signs (21). Such delays, especially in the era of COVID-19, can pose risks to patient survival. Newly published guidelines caused hesitation in both healthcare personnel and patients regarding continuing cancer treatments, screenings, and prescribing treatment for new cases (20, 44). Overwhelmed healthcare systems reduced face-to-face interactions, and rising fear among cancer patients population did not help to reduce hesitation. One study in England showed that registration for SACT was reduced in the wake of COVID-19 in April 2020 compared to prior months (45). Of note, the pandemic introduced new perspectives and broadened the discussion of introducing SACT to compromised individuals in a time of global illness, fear, and reluctance. During COVID-19, the decision-making process for choosing SACT for cancer treatment prompted further discussion about the careful consideration and evaluation of the balance between toxic regimens and palliative care with overall benefit and whether or not such treatments have an indication (46).

The pandemic has also opened more studies into the costs of delayed diagnosis and treatment for certain cancers. In a study of 20 invasive cancer types, lung cancer in particular, in addition to bladder, liver, and stomach cancer, causes a relatively high reduction in 10-yr net survival across all age groups, particularly those who are <70 years old, when the diagnosis is delayed by 3 years. Compared to the urgent investigatory referral for lung cancer, patients who delay referral by 2 months may result in an estimated 0.0–0.7 life-years loss on average. Specifically, urgent investigation can cause a relatively high per-patient net survival gain in patients with lung cancer (21).

## Conclusion

The overall survival of patients with lung cancer who present with malignant pericardial effusion has been reported in the literature to be as short as four months However, the addition of novel therapies such as immunotherapy might alter this grim prognosis, as shown in this study. Future larger prospective studies are needed to better evaluate the clinical outcome of patients with malignant pericardial effusion.

## Data Availability Statement

The raw data supporting the conclusions of this article will be made available by the authors, without undue reservation.

## Ethics Statement

Written informed consent was obtained from the individual(s) for the publication of any potentially identifiable images or data included in this article.

## Author Contributions

All authors listed have made a substantial, direct, and intellectual contribution to the work and approved it for publication.

## Conflict of Interest

The authors declare that the research was conducted in the absence of any commercial or financial relationships that could be construed as a potential conflict of interest.

## Publisher’s Note

All claims expressed in this article are solely those of the authors and do not necessarily represent those of their affiliated organizations, or those of the publisher, the editors and the reviewers. Any product that may be evaluated in this article, or claim that may be made by its manufacturer, is not guaranteed or endorsed by the publisher.

## References

[1] Ben-Horin S Bank I Guetta V Livneh A. Large Symptomatic Pericardial Effusion as the Presentation of Unrecognized Cancer: A Study in 173 Consecutive Patients Undergoing Pericardiocentesis. Med (Baltimore) (2006) 85(1):49–53. doi: 10.1097/01.md.0000199556.69588.8e 16523053

[2] Corey GR Campbell PT Van Trigt P Kenney RT O'Connor CM Sheikh KH. Etiology of Large Pericardial Effusions. Am J Med (1993) 95(2):209–13. doi: 10.1016/0002-9343(93)90262-N 8356985

[3] Posner MR Cohen GI Skarin AT. Pericardial Disease in Patients With Cancer. The Differentiation of Malignant From Idiopathic and Radiation-Induced Pericarditis. Am J Med (1981) 71(3):407–13. doi: 10.1016/0002-9343(81)90159-5 7282729

[4] Buck M Ingle JN Giuliani ER Gordon JR Therneau TM. Pericardial Effusion in Women With Breast Cancer. Cancer (1987) 60(2):263–9. doi: 10.1002/1097-0142(19870715)60:2<263::AID-CNCR2820600225>3.0.CO;2-N 3594362

[5] Klatt EC Heitz DR. Cardiac Metastases. Cancer (1990) 65(6):1456–9. doi: 10.1002/1097-0142(19900315)65:6<1456::AID-CNCR2820650634>3.0.CO;2-5 2306690

[6] Ou SH Zell JA. Validation Study of the Proposed IASLC Staging Revisions of the T4 and M Non-Small Cell Lung Cancer Descriptors Using Data From 23,583 Patients in the California Cancer Registry. J Thorac Oncol (2008) 3(3):216–27. doi: 10.1097/JTO.0b013e318164545d 18317063

[7] Imazio M Adler Y. Management of Pericardial Effusion. Eur Heart J (2013) 34(16):1186–97. doi: 10.1093/eurheartj/ehs372 23125278

[8] Petrofsky M. Management of Malignant Pericardial Effusion. J Adv Pract Oncol (2014) 5(4):281–9. doi: 10.6004/jadpro.2014.5.4.5 PMC4457183 26110072

[9] Verlaan D Veltman JD Grady B. Total Electrical Alternans in a Patient With Malignant Pericardial Tamponade. BMJ Case Rep (2018) 2018. doi: 10.1136/bcr-2018-224771 PMC6058104 30030246

[10] Eisenberg MJ de Romeral LM Heidenreich PA Schiller NB Evans GT Jr. The Diagnosis of Pericardial Effusion and Cardiac Tamponade by 12-Lead ECG. A Technol assessment Chest (1996) 110(2):318–24. doi: 10.1378/chest.110.2.318 8697827

[11] Meyers DG Bagin RG Levene JF. Electrocardiographic Changes in Pericardial Effusion. Chest (1993) 104(5):1422–6. doi: 10.1378/chest.104.5.1422 8222799

[12] Adler Y Charron P Imazio M Badano L Baron-Esquivias G Bogaert J. ESC Guidelines for the Diagnosis and Management of Pericardial Diseases: The Task Force for the Diagnosis and Management of Pericardial Diseases of the European Society of Cardiology (ESC)Endorsed by: The European Association for Cardio-Thoracic Surgery (EACTS). Eur Heart J (2015) 36(42):2921–64. doi: 10.1093/eurheartj/ehv318 PMC7539677 26320112

[13] Nguyen O Ouellette D. Survival Post Surgery for Malignant Pericardial Effusion. Clin Pract (2011) 1(2):e38. doi: 10.4081/cp.2011.e38 24765299 PMC3981241

[14] Hu ZG Hu K Li WX Zeng FJ. Prognostic Factors and Nomogram for Cancer-Specific Death in Non Small Cell Lung Cancer With Malignant Pericardial Effusion. PloS One (2019) 14(5):e0217007. doi: 10.1371/journal.pone.0217007 31095610 PMC6521987

[15] Haddad DE Iliescu C William WN Mouhayar E. Survival Outcome of Cancer Patients With Pericardial Effusions. J Clin Oncol (2015) 33(15):9573–. doi: 10.1200/jco.2015.33.15_suppl.9573

[16] Gross JL Younes RN Deheinzelin D Diniz AL da Silva RA Haddad FJosé. Surgical Management of Symptomatic Pericardial Effusion in Patients With Solid Malignancies. Ann Surg Oncol (2006) 13:1732–8. doi: 10.1245/s10434-006-9073-1 17028771

[17] Campione A Cacchiarelli M Ghiribelli C Caloni V D’Agata A Gotti G. Which Treatment in Pericardial Effusion? J Cardiovasc Surg (Torino) (2002) 43(5):735–9. 12386594

[18] Vakamudi S Ho N Cremer PC. Pericardial Effusions: Causes, Diagnosis, and Management. Prog Cardiovasc Dis (2017) 59(4):380–8. doi: 10.1016/j.pcad.2016.12.009 28062268

[19] Kallianpur AA Samra SS Nimbran V Gupta R Akkarappatty C Kallianpur AA. Pericardial-Peritoneal Window: A Novel Palliative Treatment for Malignant and Recurrent Cardiac Tamponade. Indian J Palliat Care (2013) 19(2):116–8. doi: 10.4103/0973-1075.116710 PMC3775022 24049355

[20] Curigliano G Banerjee S Cervantes A Garassino MC Garrido P Girard N. Managing Cancer Patients During the COVID-19 Pandemic: An ESMO Multidisciplinary Expert Consensus. Ann Oncol (2020) 31(10):1320–35. doi: 10.1016/j.annonc.2020.07.010 PMC7836806 32745693

[21] Sud A Torr B Jones ME Broggio J Scott S Loveday C. Effect of Delays in the 2-Week-Wait Cancer Referral Pathway During the COVID-19 Pandemic on Cancer Survival in the UK: A Modelling Study. Lancet Oncol (2020) 21(8):1035–44. doi: 10.1016/S1470-2045(20)30392-2 PMC7116538 32702311

[22] Buzaid AC Garewal HS Greenberg BR. Managing Malignant Pericardial Effusion. West J Med (1989) 150(2):174–9. PMC1026330 2471362

[23] Tsang TS Seward JB Barnes ME Bailey KR Sinak LJ Urban LH. Outcomes of Primary and Secondary Treatment of Pericardial Effusion in Patients With Malignancy. Mayo Clin Proc (2000) 75(3):248–53. doi: 10.4065/75.3.248 10725950

[24] Lestuzzi C. Neoplastic Pericardial Disease: Old and Current Strategies for Diagnosis and Management. World J Cardiol (2010) 2(9):270–9. doi: 10.4330/wjc.v2.i9.270 PMC2999066 21160603

[25] Martinoni A Cipolla CM Cardinale D Civelli M Lamantia G Colleoni M. Long-Term Results of Intrapericardial Chemotherapeutic Treatment of Malignant Pericardial Effusions With Thiotepa. Chest (2004) 126(5):1412–6. doi: 10.1378/chest.126.5.1412 15539706

[26] Celik S Lestuzzi C Cervesato E Dequanter D Piotti P De Biasio M. Systemic Chemotherapy in Combination With Pericardial Window has Better Outcomes in Malignant Pericardial Effusions. J Thorac Cardiovasc Surg (2014) 148(5):2288–93. doi: 10.1016/j.jtcvs.2014.04.031 24836991

[27] Kim SH Kwak MH Park S Kim HJ Lee HS Kim MS. Clinical Characteristics of Malignant Pericardial Effusion Associated With Recurrence and Survival. Cancer Res Treat (2010) 42(4):210–6. doi: 10.4143/crt.2010.42.4.210 PMC3021740 21253323

[28] Harada K Ogasawara M Shido A Meno A Oda S Yoshida S. Pericardial Tamponade During Pembrolizumab Treatment in a Patient With Advanced Lung Adenocarcinoma: A Case Report and Review of the Literature. Thorac Cancer (2020) 11(5):1350–3. doi: 10.1111/1759-7714.13399 PMC7180565 32181993

[29] Shaheen S Mirshahidi H Nagaraj G Hsueh CT. Conservative Management of Nivolumab-Induced Pericardial Effusion: A Case Report and Review of Literature. Exp Hematol Oncol (2018) 7:11. doi: 10.1186/s40164-018-0104-y 29761026 PMC5941729

[30] Nesfeder J Elsensohn AN Thind M Lennon J Domsky S. Pericardial Effusion With Tamponade Physiology Induced by Nivolumab. Int J Cardiol (2016) 222:613–4. doi: 10.1016/j.ijcard.2016.08.023 27517649

[31] Kushnir I Wolf I. Nivolumab-Induced Pericardial Tamponade: A Case Report and Discussion. Cardiology (2017) 136(1):49–51. doi: 10.1159/000447053 27554835

[32] Vittorio A Sharma R Siejka D Bhattarai K Hardikar A. Recurrent Pericardial Effusion While Receiving Nivolumab for Metastatic Lung Adenocarcinoma: Case Report and Review of the Literature. Clin Lung Cancer (2018) 19(5):e717–e20. doi: 10.1016/j.cllc.2018.05.010 29937384

[33] Kolla BC Patel MR. Recurrent Pleural Effusions and Cardiac Tamponade as Possible Manifestations of Pseudoprogression Associated With Nivolumab Therapy- a Report of Two Cases. J Immunother Cancer (2016) 4:80. doi: 10.1186/s40425-016-0185-2 27895919 PMC5109681

[34] Kazandjian D Suzman DL Blumenthal G Mushti S He K Libeg M. FDA Approval Summary: Nivolumab for the Treatment of Metastatic Non-Small Cell Lung Cancer With Progression On or After Platinum-Based Chemotherapy. Oncologist (2016) 21(5):634–42. doi: 10.1634/theoncologist.2015-0507 PMC4861371 26984449

[35] The New York Times. Coronavirus World Map: Tracking the Global Outbreak: The New York Times (2021). Available at: https://www.nytimes.com/interactive/2020/world/coronavirus-maps.html.

[36] Rogado J Pangua C Serrano-Montero G Obispo B Marino AM Perez-Perez M. Covid-19 and Lung Cancer: A Greater Fatality Rate? Lung Cancer (2020) 146:19–22. doi: 10.1016/j.lungcan.2020.05.034 32505076 PMC7260554

[37] Saini KS Tagliamento M Lambertini M McNally R Romano M Leone M. Mortality in Patients With Cancer and Coronavirus Disease 2019: A Systematic Review and Pooled Analysis of 52 Studies. Eur J Cancer (2020) 139:43–50. doi: 10.1016/j.ejca.2020.08.011 32971510 PMC7467090

[38] Zhang L Zhu F Xie L Wang C Wang J Chen R. Clinical Characteristics of COVID-19-Infected Cancer Patients: A Retrospective Case Study in Three Hospitals Within Wuhan, China. Ann Oncol (2020) 31(7):894–901. doi: 10.1016/j.annonc.2020.03.296 32224151 PMC7270947

[39] Liang W Guan W Chen R Wang W Li J Xu K. Cancer Patients in SARS-CoV-2 Infection: A Nationwide Analysis in China. Lancet Oncol (2020) 21(3):335–7. doi: 10.1016/S1470-2045(20)30096-6 PMC7159000 32066541

[40] Calles A Aparicio MI Alva M Bringas M Gutierrez N Soto J. Outcomes of COVID-19 in Patients With Lung Cancer Treated in a Tertiary Hospital in Madrid. Front Oncol (2020) 10:1777. doi: 10.3389/fonc.2020.01777 33042826 PMC7525070

[41] Bonomi M Maltese M Brighenti M Muri M Passalacqua R. Tocilizumab for COVID-19 Pneumonia in a Patient With Non-Small-Cell Lung Cancer Treated With Chemoimmunotherapy. Clin Lung Cancer (2021) 22(1):e67–e9. doi: 10.1016/j.cllc.2020.08.002 PMC7448773 32952047

[42] Chakraborty C Sharma AR Sharma G Bhattacharya M Lee SS. SARS-CoV-2 Causing Pneumonia-Associated Respiratory Disorder (COVID-19): Diagnostic and Proposed Therapeutic Options. Eur Rev Med Pharmacol Sci (2020) 24(7):4016–26. doi: 10.26355/eurrev_202004_20871 32329877

[43] Gattinoni L Chiumello D Caironi P Busana M Romitti F Brazzi L. COVID-19 Pneumonia: Different Respiratory Treatments for Different Phenotypes? Intensive Care Med (2020) 46(6):1099–102. doi: 10.1007/s00134-020-06033-2 PMC7154064 32291463

[44] Hanna TP Evans GA Booth CM. Cancer, COVID-19 and the Precautionary Principle: Prioritizing Treatment During a Global Pandemic. Nat Rev Clin Oncol (2020) 17(5):268–70. doi: 10.1038/s41571-020-0362-6 PMC7117554 32242095

[45] Clark JJ Dwyer D Pinwill N Clark P Johnsonk P Hacksaw A. The Effect of Clinical Decision Making for Initiation of Systemic Anticancer Treatments in Response to the COVID-19 Pandemic in England: A Retrospective Analysis. Lancet Oncol (2021) 22(1):66–73. doi: 10.1016/S1470-2045(20)30619-7 33253639 PMC7833889

[46] van de Haar J Hoes LR Coles CE Seamon K Fröhling S Jäger D. Caring for Patients With Cancer in the COVID-19 Era. Nat Med (2020) 26(5):665–71. doi: 10.1038/s41591-020-0874-8 32405058

